# Skeletal Muscle and Bone – Emerging Targets of Fibroblast Growth Factor-21

**DOI:** 10.3389/fphys.2021.625287

**Published:** 2021-03-08

**Authors:** Hui Sun, Matthew Sherrier, Hongshuai Li

**Affiliations:** ^1^Musculoskeletal Growth & Regeneration Laboratory, Department of Orthopaedic Surgery, University of Pittsburgh, Pittsburgh, PA, United States; ^2^Department of Orthopaedic Surgery, Shanghai Jiao Tong University Affiliated Sixth People’s Hospital, Shanghai, China; ^3^Department of Physical Medicine and Rehabilitation, University of Pittsburgh Medical Center, Pittsburgh, PA, United States

**Keywords:** skeletal muscle, fibroblast growth factor 21, bone, myokine, expression, muscular dystrophy, Osteoporosis

## Abstract

Fibroblast growth factor 21 (FGF21) is an atypical member of the FGF family, which functions as a powerful endocrine and paracrine regulator of glucose and lipid metabolism. In addition to liver and adipose tissue, recent studies have shown that FGF21 can also be produced in skeletal muscle. As the most abundant tissue in the human body, skeletal muscle has become increasingly recognized as a major site of metabolic activity and an important modulator of systemic metabolic homeostasis. The function and mechanism of action of muscle-derived FGF21 have recently gained attention due to the findings of considerably increased expression and secretion of FGF21 from skeletal muscle under certain pathological conditions. Recent reports regarding the ectopic expression of FGF21 from skeletal muscle and its potential effects on the musculoskeletal system unfolds a new chapter in the story of FGF21. In this review, we summarize the current knowledge base of muscle-derived FGF21 and the possible functions of FGF21 on homeostasis of the musculoskeletal system with a focus on skeletal muscle and bone.

## Introduction

The fibroblast growth factor (FGF) family is a group of 22 related proteins grouped into six subfamilies, based on genetic and functional similarities, that have a wide variety of functions (Degirolamo et al., 2016). FGF21, together with FGF19 (human ortholog of mouse FGF15) and FGF23, belongs to the FGF19 subfamily, which represents an atypical group of FGFs due to the lack of affinity for heparin sulfates that allows them to act in an endocrine manner to influence the enterohepatic circulation of bile, regulate glucose and lipid metabolism, and maintain phosphorus and calcium homeostasis (Dolegowska et al., 2019). FGF15/19 is produced in the liver in response to the postprandial release of bile acids (Struik et al., 2019), fat-soluble vitamins A and D, and cholesterol (Schmidt et al., 2010; Henkel et al., 2011) and primarily functions as a negative feedback mechanism to decrease bile acid synthesis (Holt et al., 2003). In addition to controlling the enterohepatic circulation of bile acid, FGF15/19 also regulates systemic lipid and glucose metabolism *via* its action on the liver, adipose tissue, and central nervous system (Owen et al., 2015; Izaguirre et al., 2017). FGF23 is mainly produced in osteocytes and functions as an important regulator of phosphate and calcium metabolism through multiple organs, especially the kidney (Edmonston and Wolf, 2020). FGF21 is primarily produced by the liver and adipose tissue in response to various metabolic, oxidative, nutritional, hormonal, or environmental stimuli, which provides signaling to multiple tissues including the central nervous system (Bookout et al., 2013) and adipose tissue (Canto and Auwerx, 2012) to mediate carbohydrate and lipid metabolism (Kim and Lee, 2014; Markan et al., 2014).

Fibroblast growth factor 21 functions not only as a regulator of energy metabolism, but also as a stress hormone for maintenance of tissue homeostasis in an autocrine, paracrine, or endocrine fashion (Kim and Lee, 2014; Salminen et al., 2017a). Along these lines, FGF21 expression is induced by the integrated stress response (ISR) pathway, an evolutionarily conserved adaptive system of eukaryotic cells for the restoration of cellular homeostasis in response to diverse stimuli including aging, obesity, and nutritional stressors (Salminen et al., 2017a). The cellular context in addition to the character and intensity of the stressful precipitant dictate the outcome of the ISR (Pakos-Zebrucka et al., 2016). As such, the beneficial or detrimental effects of FGF21 are dependent on an integration of variables, making this unique and controversial hormone functional therapeutically and as a biomarker of disease (Oost et al., 2020). Although complex in its function and regulation, the current integrative physiological role of FGF21 is as a key regulator in the adaptation to stress that can limit the progression of metabolic disease states with the goal of restoring homeostasis (Kim and Lee, 2014).

In addition to the hepatic and adipose production, recent studies have demonstrated that FGF21 can be expressed and secreted from other peripheral tissues, such as skeletal muscle (Keipert et al., 2014; Fisher and Maratos-Flier, 2016; Lehtonen et al., 2016; Tezze et al., 2019), thymus (Youm et al., 2016), and pancreas (Nishimura et al., 2000; Fisher and Maratos-Flier, 2016). In humans, skeletal muscle is the most abundant tissue in the body, accounting for more than 40% of body weight in healthy individuals (Wang and Pessin, 2013), and has become increasingly recognized as a major site of metabolic activity and an important modulator of systemic metabolic homeostasis (Tezze et al., 2017). Growing evidence suggests that muscle-derived growth factors or cytokines, known as myokines (Pedersen and Febbraio, 2012), may be responsible for the endocrine effects (Demontis et al., 2013). The function and mechanisms of action of muscle-derived FGF21 have drawn attention due to the findings of considerable amounts of FGF21 expressed and secreted under certain pathologic conditions (Lehtonen et al., 2016). Despite recent publications on muscle-derived FGF21 and its effect on the musculoskeletal system, significant knowledge gaps exist. The purpose of this review is to summarize the current knowledge base of muscle-derived FGF21 and the possible functions of FGF21 on homeostasis of the musculoskeletal system with a focus on skeletal muscle and bone. Knowledge of the ectopic expression of FGF21 from skeletal muscle and its potential effects on the musculoskeletal system has provided new avenues of investigation into the relevance of FGF21 to health and disease.

## FGF21 as a Myokine

Myokines are cytokines or peptides synthesized and released by muscle in response to muscular contraction or various stimuli (Pedersen et al., 2007). Under basal conditions, the expression of FGF21 is predominantly from liver and adipose tissue (Nishimura et al., 2000), however, the expression and secretion of FGF21 from skeletal muscle is significantly increased under certain conditions, such as mitochondrial dysfunction (Keipert et al., 2014; Lehtonen et al., 2016; Steele et al., 2016; Khan et al., 2017; Romanello et al., 2019), muscular dystrophy (Lovadi et al., 2017; Zhou et al., 2018; Li et al., 2020), and exercise (Ost et al., 2016; Kruse et al., 2017; Morville et al., 2018). Thus, in addition to being a hepatokine and adipokine, FGF21 is also well-established as a myokine (Pedersen and Febbraio, 2012; Itoh, 2014; Pereira et al., 2017). In this section, we will discuss the current knowledge base regarding the identification of muscle-derived FGF21 and the mechanisms that drive its expression from skeletal muscle.

### Mitochondrial Disorders

Fibroblast growth factor 21 is induced in and secreted from skeletal muscle in mitochondrial myopathies and insults of various stresses in skeletal muscle. Increased levels of FGF21 in the skeletal muscle and serum have been demonstrated in mouse models of familial progressive external ophthalmoplegia, a progressive adult-onset mitochondrial respiratory chain deficiency (Badenhorst et al., 2015), skeletal muscle-specific ablation of autophage-related 7 (Kim et al., 2013a), and skeletal muscle specific optic atrophy 1 (OPA1, a mitochondrial fusion protein) deficiency (Pereira et al., 2017; Tezze et al., 2017; Rodriguez-Nuevo et al., 2018), all of which result in mitochondrial dysfunction. Additionally, overexpression of uncoupling protein 1 (UCP-1), a key regulatory molecule of mitochondrial function results in the ectopic expression of FGF21 from skeletal muscle (Keipert et al., 2014). Impaired mitochondrial fat oxidation has also been demonstrated to induce the expression of FGF21 in skeletal muscle. The transgenic overexpression of perilipin 5 (a lipid droplet protein), which can increase lipid storage in the muscle and in turn affect its utilization as an energy source by skeletal muscle, stimulates the expression of FGF21 from skeletal muscle (Harris et al., 2015). Inversely, perilipin 5 deletion increases fatty acid oxidation and decreases FGF21 production by muscle (Montgomery et al., 2018). Skeletal muscle-specific deletion of carnitine palmitoyltransferase-1b, which transports long-chain fatty acid into mitochondria for beta-oxidation, also induces FGF21 expression from muscle (Vandanmagsar et al., 2016).

In humans, serum FGF21 levels are significantly increased in patients with primary muscle-manifesting respiratory chain deficiencies, particularly those caused by pathogenic mutations in mitochondrial DNA (Suomalainen et al., 2011; Crooks et al., 2014) with the muscle believed to be the primary contributory organ to circulating levels (Crooks et al., 2014). Thus, FGF21 has recently gained attention as a potential biomarker of mitochondrial diseases (Tyynismaa et al., 2010; Suomalainen et al., 2011; Lehtonen et al., 2016, 2020) and could represent a potential target for the treatment of mitochondrial myopathies and muscle mitochondria dysfunction.

### Muscular Dystrophy and Muscle Regeneration

Elevated serum FGF21 has been demonstrated in animal models of Duchenne muscular dystrophy (DMD; Zhou et al., 2018; Li et al., 2020) and is primarily derived from dystrophic muscle (Li et al., 2020). However, the mechanisms that drive the expression of FGF21 from dystrophic skeletal muscle is still largely unknown. Mitochondrial deficiency (Timpani et al., 2015), autophage dysfunction (De Palma et al., 2012), and endoplasmic reticulum (ER) stressors (Pauly et al., 2017) have been implicated as part of the pathogenesis of DMD and have also been shown to increase the expression of FGF21 from skeletal muscle (Zhou et al., 2018; Li et al., 2020). Furthermore, one of the hallmarks of DMD pathology is constant muscle degeneration and regeneration (Rosenberg et al., 2015). Interestingly, FGF21 expression has been detected in C2C12 cells during myogenic differentiation, and myoblast determination protein 1 (MyoD) is implicated as a major controller of FGF21 gene transcription (Ribas et al., 2014). Thus, it is possible that higher expression of FGF21 in DMD may be due to increased myogenic differentiation. Further studies are needed to verify if elevated FGF21 is also present in human patients, to elucidate the mechanism behind the increased FGF21 from dystrophic muscle, and to determine whether downregulation of FGF21 is accompanied by an improved muscle function.

### Exercise

Multiple studies have shown that FGF21 is associated with exercise, however, the literature regarding the exercise-induced changes in FGF21 in the serum, liver, and skeletal muscle is inconsistent and contradictory. With regards to the serum levels, studies in mice and humans have shown an exercise-induced increase (Cuevas-Ramos et al., 2012; Kim et al., 2013b), decrease (Yang et al., 2011; Taniguchi et al., 2016; Shabkhiz et al., 2020), or no change in circulating FGF21 (Andersen et al., 2014; Besse-Patin et al., 2014). Additionally, the data regarding the impact of liver-derived FGF21 during exercise are contradictory. Hansen et al. (2015, 2016) have shown an induction of hepatic FGF21 synthesis in response to exercise through the ATF4/PPARα mediated pathway, glucagon to insulin ratio, and free fatty acid levels. Furthermore, acute and long-term endurance exercise at intensities between 50 and 80% VO2max in humans results in elevated serum levels of FGF21 *via* increased hepatic expression of FGF21 but without increased expression in skeletal muscle or adipose tissue (Cuevas-Ramos et al., 2012). Similarly, a mouse study has shown an exercise-induced increase in hepatic FGF21 expression (Berglund et al., 2011). However, other studies have shown no increase in hepatic FGF21 after exercise (Fletcher et al., 2016; Loyd et al., 2016). It is important to note that there are significant methodological issues that likely account for the inconsistencies. Firstly, the mice and humans studied were in various metabolic states, including exercise-trained vs. untrained, type 2 diabetes mellitus, obesity, and advanced age. Secondly, exercise protocols were not comparable with variations in the types, intensities, and duration of exercise. Finally, FGF21 expression could also be affected by the participant’s diet and circadian rhythm (Yu et al., 2011) in addition to the interspecies variability (Staiger et al., 2017; Keuper et al., 2020). Future studies should address and control for these important confounding variables.

Recent studies have raised the question of how significant the contribution of exercise-induced skeletal muscle-derived FGF21 is to bioactive and circulatory levels. Previous studies demonstrated that resistance training and higher intensity exercise increases FGF21 expression in skeletal muscle (Tanimura et al., 2016; Sabaratnam et al., 2018), although to a lesser extent than the increase in hepatic expression. However, recent reports have challenged this notion. Parmar et al. (2018) demonstrated no change in skeletal muscle-derived FGF21 between baseline and 48h following a single-leg maximal eccentric contraction exercise. Additionally, moderate-intensity continuous training has been recently shown to produce higher expression of FGF21 and β-klotho expression in the liver and muscle of the obese mice than high-intensity interval training (Xiong et al., 2020). As mentioned, the contradictory data and inconsistencies in the literature can be attributed to variation in study design and outcome measures. The source of FGF21 during exercise is likely dependent on the individual training level (i.e., athlete vs. untrained), intensity, type, and duration of the exercises along with the time points at which blood samples are collected. More studies are needed to understand how and to what degree exercise affects the expression of FGF21 from the liver and skeletal muscle. Overall, it is commonly recognized that the exercise influences the systemic, hepatic, and skeletal muscle-derived FGF21, although the mechanism remains unknown.

### Aging

Although early studies implicated FGF21 as a pro-longevity factor, recent research has questioned that notion. Extended lifespan has been observed in a transgenic mouse line, which expressed the FGF21 gene (Tg-FGF21) from liver under the control of an ApoE promoter (Inagaki et al., 2008; Zhang et al., 2012). There are also indications that hepatic overexpression of FGF21 can protect against age-related immune senescence (Youm et al., 2016). Youm et al. (2016) observed that thymic involution, a common hallmark of aging, was significantly delayed in Tg-FGF21 mice. FGF21 possesses many mechanistic properties that may impact the aging process. FGF21 stimulates adenosine monophosphate-activated protein kinase (AMPK) signaling (Chau et al., 2010; Salminen et al., 2017c), an established pro-longevity pathway, both directly through the FGFR1/klotho complex and indirectly *via* induction of adiponectin expression (Lin et al., 2013; Hui et al., 2016). FGF21 can also facilitate crosstalk among hormonal systems such as the somatotropic axis and the hypothalamic-pituitary-adrenal pathway (Salminen et al., 2017b). Additionally, FGF21 has been proposed to regulate longevity through its ability to promote interactions between energy metabolism and stress responses (Salminen et al., 2017a,b).

Despite the aforementioned beneficial effects observed in experimental animal models, the notion that FGF21 is a pro-longevity factor has been challenged in the literature. Studies indicate that circulating levels of FGF21 are elevated in several metabolic diseases, such as obesity, type 2 diabetes, and fatty liver disease (Zhang et al., 2008; Liu et al., 2015). FGF21 has been shown to increase with age among healthy individuals independent of body composition, e.g., fat percent and body mass index (Hanks et al., 2015). Interestingly, a recent study has found that circulating levels of FGF21 are associated with worsened health parameters and mortality in the elderly (Conte et al., 2018). In these contexts, whether FGF21 is beneficial or detrimental is still debated. Moreover, the source organ or tissue of elevated circulating FGF21 during aging is still unclear.

It remains unknown what role skeletal muscle-derived FGF21 plays in its potential health and life extension effects. Skeletal muscle has been emerging as an important mediator of systemic metabolic homeostasis (Baskin et al., 2015) and myokines are likely, in part, responsible for the modulation of aging physiology (Demontis et al., 2013). There is mounting evidence indicating that the elevated FGF21 expression from skeletal muscle under a variety of stresses can regulate whole-body metabolism as evidenced by preventing diet-induced obesity and insulin resistance (Kim et al., 2013a; Jung et al., 2015; Pereira et al., 2017). However, whether this ectopic expression of muscle-derived FGF21 imparts a beneficial effect on longevity remains unknown. Interestingly, Tezze et al. (2017) reported that the increased expression and secretion of FGF21 from skeletal muscle in a muscle-specific deletion of the OPA1 mouse model appears to be responsible for an accelerated aging phenotype. In this study, increased expression of myokine FGF21 was correlated with a precocious systemic senescence phenotype and premature death, while inhibition of FGF21 greatly ameliorated the aging phenotype (Tezze et al., 2017). Given that pro-longevity effects have mainly been observed in transgenic mice with hepatic FGF21 overexpression (Inagaki et al., 2008; Zhang et al., 2012; Youm et al., 2016), this study highlights the possibility that the longevity effects of FGF21 may be liver-specific. Further studies using tissue-specific over/down the expression of FGF21 are needed to determine whether the longevity effects of FGF21 are origin tissue-specific and to gain mechanistic insights.

### Nutrient Stress

A growing body of literature has demonstrated that nutritional stressors and dietary macronutrient composition resulting in metabolically unhealthy obesity can regulate FGF21 expression and signaling, serving to coordinate and restore metabolic homeostasis. In this section, we highlight fasting and obesity as two ends of the nutritional spectrum with a focus what is known about the role of skeletal muscle-derived FGF21.

Fasting in mice and humans induces expression of hepatic FGF21 *via* the peroxisome proliferator-activated receptor *α* (PPARα) pathway (Inagaki et al., 2007; Galman et al., 2008). This PPARα-mediated FGF21 induction also increases fatty acid oxidation and ketogenesis in the setting of nutritional ketosis, suggesting that FGF21 functions in the adaptation to fasting or ketosis (Badman et al., 2007). Whereas the induction in FGF21 occurs within 24h of fasting in mice (Badman et al., 2007), elevations in FGF21 in humans are not seen in short-term fasting regimens (Galman et al., 2008; Dushay et al., 2010; Fazeli et al., 2015b; Nygaard et al., 2018; Vinales et al., 2019) but only appear after prolonged fasting of at least 7days (Galman et al., 2008; Fazeli et al., 2015b). The higher metabolic rate of mice as compared with humans has been proposed as an explanation for this discrepancy. With regards to skeletal-muscle derived FGF21, in 24-h fasted WT mice, the levels of FGF21 mRNA were significantly increased (Oost et al., 2019). However, the contribution of FGF21 as a myokine to the adaptive starvation response in mice or humans remains unknown.

Nutrient overload and obesity are also capable of influencing gene expression and circulating levels of FGF21 in mice and humans (Zhang et al., 2008; Fisher et al., 2010). Studies have demonstrated impaired FGF21 signaling in the liver, pancreas, and white adipose tissue of obese mice (Fisher et al., 2010; So et al., 2013), initially suggesting that obesity is an FGF21-resistant state. However, the concept of FGF21 resistance remains incompletely understood as a result of the undetermined overlap and differences between the physiological and pharmacological effects of FGF21 in addition to its mechanisms of action on different tissues (Hale et al., 2012; Markan, 2018; Martinez-Garza et al., 2019). The current understanding is that the liver is the primary contributory organ of circulating FGF21in the setting of metabolically unhealthy obesity with unelucidated impact of FGF21 as a myokine to the systemic milieu (Keuper et al., 2020).

### Signaling Pathways That Drive the Expression of FGF21 From Skeletal Muscle

**Figure 1:**
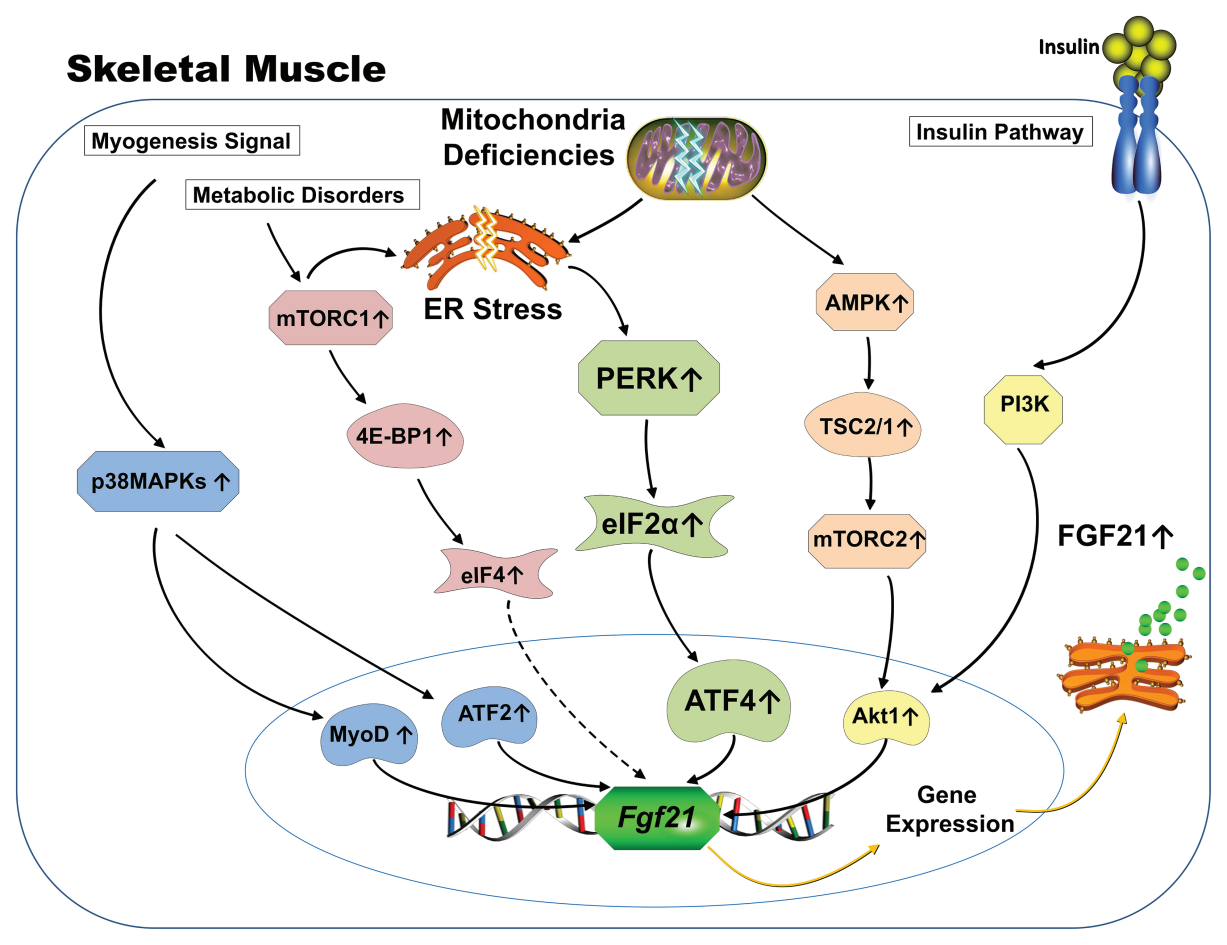
Schematic representation of known signaling pathways involved in FGF21 expression from skeletal muscle. Under physiological conditions, the expression of FGF21 from skeletal muscle is responsive to insulin stimulation *via* PI3K-Akt1 signaling pathway (yellow). FGF21 expression from skeletal muscle in various mitochondrial deficiency animal models is mainly driven by the activation of integrative stress response (green). Specifically, mitochondrial deficiency causes ER stresses, which leads to the phosphorylation of eIF2a *via* activation of PERK arm. Phosphorylation of elf2a increases ATF4, which subsequently augments transcription of the FGF21 gene. AMPK-Akt1 signaling pathway drives the FGF21 expression from skeletal muscle when mitochondrial fat oxidation is inhibited (orange). The p38 MAPK/AFT2/MyoD signaling pathway is involved in FGF21 expression during myogenesis (blue). Increased FGF21 expression from 4E-BP1 activated skeletal muscle was discovered and the 4E-BP is one of the key downstream substrates of the mTORC1 complex (red). (Dotted line represents unknown mechanisms) ER: Endoplasmic reticulum; PI3K: Phosphoinositide 3-kinase; Akt: Protein kinase B; eIF: eukaryotic translation initiation factor; PERK: Protein kinase R (PKR)-like endoplasmic reticulum kinase; ATF: Activating transcription factor; AMPK: Adenosine monophosphate activated protein kinase; MAPK: Mitogen-activated protein kinases; MyoD: Myoblast determination protein 1; 4E-BP: eukaryotic translation initiation factor 4E-binding protein; mTORC: mammalian target of rapamycin; TSC: Tuberous sclerosis complex.

Different from being canonically produced by the liver and adipose tissue in response to starvation, which is largely controlled by PPARα (Badman et al., 2007; Lundasen et al., 2007) and PPARγ (Muise et al., 2008; Wang et al., 2008), respectively, the ectopic expression of FGF21 from skeletal muscle is driven by various stress-related signaling pathways (Figure 1). Induction of activating transcription factor 4 (ATF4) as a master regulator of the ISR leads to FGF21 expression (Kim et al., 2013a; Keipert et al., 2014; Harris et al., 2015; Miyake et al., 2016) and appears to be a common link among the ER stress produced in mitochondrial deficiency and impaired autophagy mouse models. In addition, insulin stimulates the expression of FGF21 from skeletal muscle *via* the Phosphoinositide 3-kinase/Protein kinase B (PI3K/Akt1) signaling pathway (Izumiya et al., 2008; Vandanmagsar et al., 2016). The p38 mitogen-APK (MAPK)/AFT2/MyoD signaling pathway is involved in FGF21 expression during myogenesis (Ribas et al., 2014). AMPK/Akt1 signaling mainly drives FGF21 expression from skeletal muscle when mitochondrial fat oxidation is inhibited (Vandanmagsar et al., 2016). Finally, mammalian target of rapamycin (mTOR) signaling pathways have also been reported to be involved in the regulation of FGF21 expression from skeletal muscle. Both activation of mTOR complex 1 (mTORC1; Guridi et al., 2015; Tsai et al., 2015) and mTORC2 (Vandanmagsar et al., 2016) have been reported to induce FGF21 expression from muscle.

## The Effects of FGF21 on Skeletal Muscle

**Figure 2:**
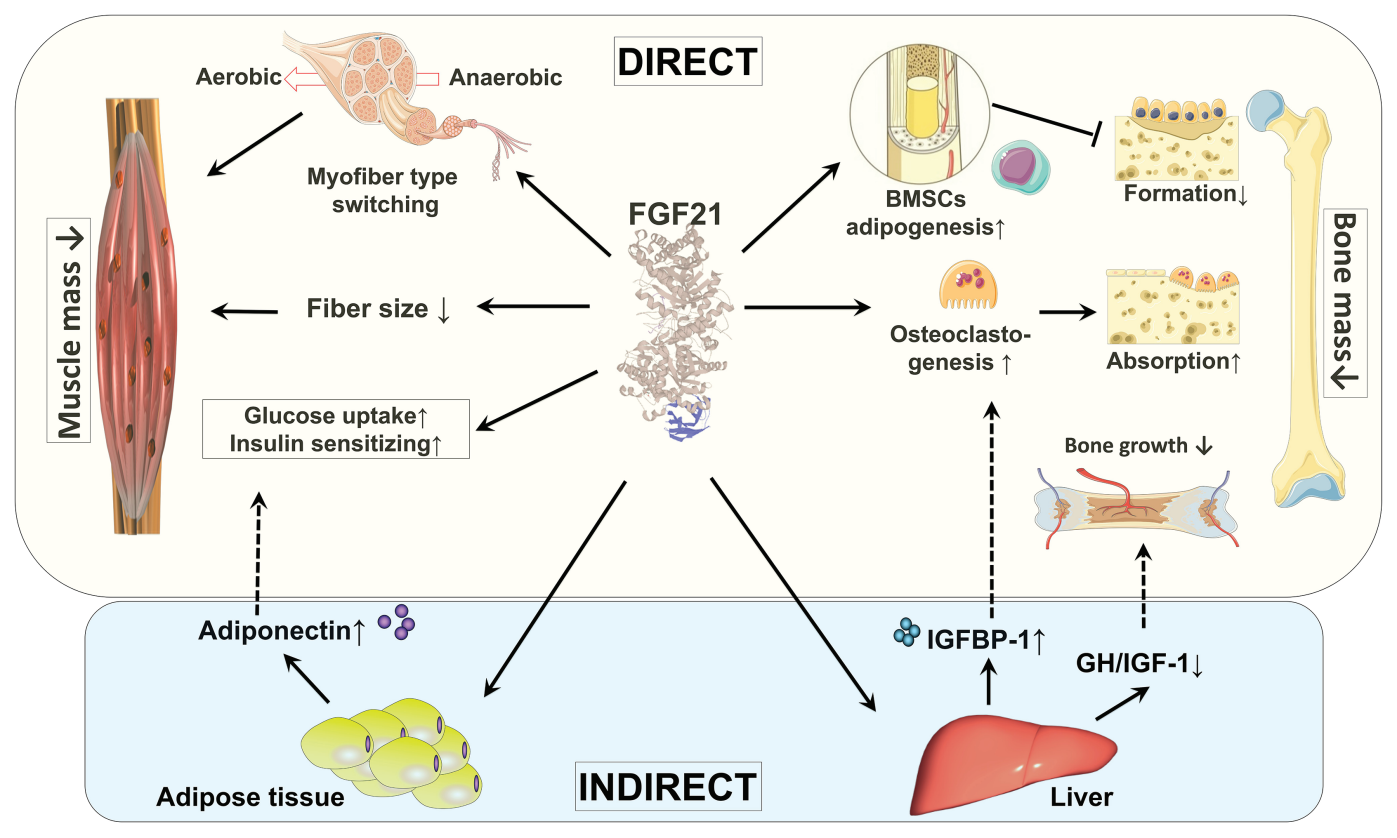
Direct and indirect effects of FGF21 on skeletal muscle and bone. By regulating fiber type distribution and fiber size, skeletal muscle mass might be impacted by FGF21. Under certain pathological conditions, FGF21 is a causative factor of muscle atrophy. The direct effects of FGF21 on skeletal muscle are enhancing glucose uptake and insulin sensitization. FGF21’s action on muscle glucose metabolism might also be indirectly mediated by adiponectin secreted from adipose tissue. By stimulation of adipogenesis in BMSCs by FGF21, bone formation is decreased. FGF21 may stimulate bone absorption directly by increasing osteoclastogenesis and indirectly *via* induction of IGFBP-1. In addition, by blunting the GH/IGF-1 signaling pathway in liver, bone growth is indirectly inhibited by FGF21. BMSCs: bone marrow-derived mesenchymal stem cells; GH/IGF-1: growth hormone/insulin-like growth factor-1; IGFBP-1: IGF-1 binding protein 1.

Although skeletal muscle was historically not considered to be a target tissue for FGF21 due to a lack of expression of β-klotho (Ito et al., 2000; Suzuki et al., 2008), recent studies have confirmed the expression of FGFRs and β-klotho in skeletal muscle albeit at very low levels (Jeon et al., 2016; Benoit et al., 2017; Tezze et al., 2017), which has opened a new area of research. In this section, we will discuss skeletal muscle as a novel target of FGF21 (Figure 2).

### The Expression of FGFRs and β-Klotho in Skeletal Muscle

Several studies have confirmed the expression of FGFRs and β-klotho in the skeletal muscle of both mice and humans (Jeon et al., 2016; Vandanmagsar et al., 2016; Benoit et al., 2017; Tezze et al., 2017) in addition to isolated myotubes in culture (Lee et al., 2012; Jeon et al., 2016). Moreover, although at a very low level compared to liver, β-klotho expression appears to be dependent on muscle fiber type, with significantly higher expression in soleus muscle (mainly slow oxidative muscle fiber) when compared to the gastrocnemius muscle (mainly fast glycolytic muscle fiber; Jeon et al., 2016).

Expression levels of β-klotho in muscle correlate with increased circulating FGF21. Lee et al. (2012) demonstrated a time-dependent increase in β-klotho expression in human skeletal muscle myotubes (HSMMs) after exposure to exogenous FGF21. Significantly elevated expression of β-klotho in muscle was also observed in a mouse model of mitochondrial fat oxidation impaired with higher induction of FGF21 (Vandanmagsar et al., 2016). Similarly, both FGF21 and β-klotho are induced in atrophic muscle after acute deletion of the mitochondrial fusion gene *Opa1* (Tezze et al., 2017). Accordingly, the specific inhibition of FGF21 in skeletal muscle is associated with downregulation of β-klotho (Tezze et al., 2017). Taken together, accumulating evidence demonstrates the coordinated regulation of FGF21 and β-klotho in skeletal muscle, which suggests their involvement in muscle homeostasis (Oost et al., 2019), although the significance remains unclear.

### Does FGF21 Affect Muscle Mass?

Few studies on FGF21 transgenic overexpression or supraphysiologic FGF21 administration in normal animal models have reported muscle phenotypes (Inagaki et al., 2008; Zhang et al., 2012; Youm et al., 2016), partially due to the early notion that skeletal muscle is an unlikely target tissue for FGF21 (Ito et al., 2000; Suzuki et al., 2008). Moreover, studies have explicitly reported the absence of noticeable signaling responses (FRS2 and ERK1/2 phosphorylation; Fisher et al., 2011) and effects on muscle mass or functionality of adult WT mice after exogenous administration of FGF21 (Benoit et al., 2017).

However, studies based on diseased animal models have suggested that skeletal muscle derived-FGF21 may be involved in the pathogenesis of muscle atrophy. Reduced muscle mass was found in UCP-1 transgenic mice (Keipert et al., 2014), carnitine palmitoyltransferase-1b specific knock-out (KO) in skeletal muscle (*Cpt1bm−/−*) mice (Vandanmagsar et al., 2016), and global and skeletal muscle conditional *Opa-1−/−* mice (Tezze et al., 2017), all of which have demonstrated elevated expression of FGF21 from skeletal muscle, as described above. Although marginal, inhibition of the elevated FGF21 expression from skeletal muscle in *Opa-1−/−* mice, *via* skeletal muscle-specific FGF21 KO, demonstrated beneficial effects on muscle mass (Tezze et al., 2017), which suggests that FGF21 might be a causative factor or mediator in the muscle atrophy observed in this mitochondrial deficiency animal model. Additionally, skeletal muscle-conditional FGF21 KO mice were significantly protected from muscle loss and weakness induced by fasting (Oost et al., 2019). Moreover, *in vivo* FGF21 overexpression from skeletal muscle *via* direct virus injections induces elevated mitophagy and results in muscle loss (Oost et al., 2019). Taken together, it is plausible that muscle-derived FGF21 plays a permissive role or mediates muscle loss in specific pathological conditions that can cause muscle atrophy. However, whether the increased expression of skeletal muscle-derived FGF21 is a causative factor of muscle atrophy or an adaptive mechanism is still unclear. Moreover, whether the effects of FGF21 on muscle atrophy is *via* binding FGFRs and β-klotho and the downstream signaling mechanisms are still unknown. To answer those questions, additional models of knock-down or KO of β-klotho expression in skeletal muscle are needed (Jensen-Cody et al., 2020).

### FGF21 in Skeletal Muscle Energy Metabolism

Fibroblast growth factor 21 is a well-established regulator of carbohydrate and lipid metabolism, which mediates the adaptive starvation response primarily *via* its action on white and brown adipose tissue and liver (Fisher and Maratos-Flier, 2016; Staiger et al., 2017; Dolegowska et al., 2019). The action of FGF21 on adipocytes results in increased insulin sensitivity, glucose uptake, fatty acid storage, and oxidative capacity (Chau et al., 2010; Staiger et al., 2017; BonDurant and Potthoff, 2018), while its action on liver results in induction of hepatic fatty acid oxidation, ketogenesis, and gluconeogenesis, as well as the suppression of *de novo* lipogenesis (Fisher and Maratos-Flier, 2016; Staiger et al., 2017). The results of FGF21-class molecule pharmacotherapy in mice, non-human primates, and humans have been critically reviewed (Sonoda et al., 2017; BonDurant and Potthoff, 2018) and include decreased body weight, blood glucose levels, insulin, triglycerides, total cholesterol, and total free fatty acids. Given that skeletal muscle is responsible for 70–80% of insulin-stimulated glucose uptake and a major determinant of glucose and lipid metabolism (Hommelberg et al., 2011; Lee et al., 2012; Bandet et al., 2019), understanding skeletal muscle as a potential target of FGF21 and the role of FGF21 in substrate metabolism remain important, unsolved questions.

The direct effect of FGF21 on skeletal muscle myotubes in enhancing glucose uptake has been demonstrated in several *in vitro* studies. Mashili et al. (2011) provided evidence that FGF21, though at a supraphysiologic dosage of 1ug/ml, had a direct effect on enhancing skeletal muscle glucose uptake in cultured HSMMs *via* increasing mRNA/protein expression of glucose transporter-1 (GLUT1) but not expression or cell surface translocation of GLUT4. Others have found that FGF21 (100 or 200ng/ml) administration increases glucose uptake in palmitate-induced insulin resistant HSMMs (Lee et al., 2012). GLUT1 and GLUT4 loss-of-function data suggest that FGF21 increases glucose uptake in HSMMs *via* not only GLUT1 but also GLUT4 (Lee et al., 2012). Additionally, studies have reported that in myotubes isolated from a carnitine palmitoyltransferase-1b skeletal muscle conditional KO mouse, FGF21 acts in a paracrine manner to increase basal glucose uptake *via* GLUT1 (Vandanmagsar et al., 2016). Thus, it appears that FGF21 acts on skeletal muscle to increase glucose uptake *via* GLUT1 (and possibly GLUT4), at least in supraphysiologic concentrations. The importance of FGF21 on skeletal muscle glucose uptake under physiological conditions is still unknown.

Studies have shown that FGF21 pre-exposure increases insulin-stimulated glucose uptake in isolated mouse soleus and extensor digitorum longus (EDL) muscles, which suggests an insulin-sensitizing effect (Mashili et al., 2011). Others demonstrated that FGF21 treatment restored palmitate-inhibited insulin signaling in HSMMs to improve the insulin sensitivity *via* phosphorylation of insulin receptor substrate 1 (IRS-1) and Akt (Lee et al., 2012). Furthermore, FGF21 can improve downstream insulin signaling in mouse skeletal muscle tissue *via* repression mTORC1, leading to subsequent repression of IRS1 phosphorylation at Ser636/639 (Vandanmagsar et al., 2016). Thus, it appears that insulin sensitization represents the primary mechanism underlying the glycemic action of FGF21.

Although several studies have demonstrated that FGF21 can regulate primary myotube glucose uptake *in vitro*, there is a dearth of research regarding the *in vivo* bioactivity of FGF21 on skeletal muscle glucose metabolism. FGF21 was found to have no impact on basal glucose uptake in isolated mouse EDL and soleus muscles (Mashili et al., 2011). In leptin-deficient (*ob/ob*) mice, a bolus injection of FGF21 increased GLUT1 mRNA in white adipose tissue, but not in skeletal muscle, liver, kidney, or brain (Kharitonenkov et al., 2005). Further *in vivo* studies are needed to validate the importance of circulating FGF21 on skeletal muscle glucose metabolism both in physiologic and pathologic conditions. In addition to its importance in the regulation of glucose metabolism, skeletal muscle also regulates lipid and ketone body metabolism (Lipina and Hundal, 2017; Sherrier and Li, 2019). However, the effects of FGF21 on lipid and ketone body metabolism in skeletal muscles are still largely unknown.

The molecular mechanism(s) of FGF21 on glucose and lipid metabolism in skeletal muscle remain unknown. Whether the effect of FGF21 is mediated *via* FGFRs/β-klotho, and which FGFR(s) play a major role has not been examined. Most studies have used whole muscle lysate for detection of β-klotho expression. Thus far, no convincing spatial histological studies have demonstrated the expression of β-klotho at a cellular level in skeletal muscle, which contains multiple cell types in addition to myofibers. Moreover, studies in mice lacking β-klotho have revealed the possibility of the existence of klotho-independent FGF21 signaling pathways whereas yet undefined co-factors are implicated (Tomiyama et al., 2010). Interestingly, a recent study reported that the metabolic effects on glucose homeostasis and insulin sensitivity of FGF21 were partially abrogated in adiponectin KO mice (Lin et al., 2013). This is exciting as adipocytes are a well-known target of FGF21 and intramuscular adipose tissue (IMAT) has emerged as an important player in insulin-resistant associated diseases; high levels of IMAT are associated with insulin resistance and loss of muscle strength (Muoio and Newgard, 2008; Muoio, 2010; Coen and Goodpaster, 2012; Addison et al., 2014). It is reasonable to speculate that this muscle/fat crosstalk *via* FGF21/adiponectin may play an important role in regulating the energy homeostasis in skeletal muscle. Furthermore, these data highlight the possibility that FGF21’s action on muscle metabolism might also be indirectly mediated by other factors.

Skeletal muscle is comprised of different fiber types, the composition and distribution of which is established during embryonic development but later can be modulated by neural and hormonal factors in addition to exercise (Schiaffino and Reggiani, 2011; Yan et al., 2011). Mature skeletal muscle is heterogeneous and composed of slow and fast-twitch fiber types, based on expression of distinct myosin heavy chain isoforms and different metabolic capabilities (Baskin et al., 2015; Talbot and Maves, 2016). Oost et al. (2019) did not find any difference in terms of fiber type distribution, fiber size, or muscle force in skeletal muscle-specific FGF21 KO mice, which suggests that skeletal muscle-derived FGF21 does not contribute to embryonic myogenesis and muscle fiber determination under normal conditions. However, studies have demonstrated that myoblasts express considerable amounts of FGF21 during myogenic differentiation (Ribas et al., 2014; Liu et al., 2017). Myoblast-derived FGF21 facilitates the switching of the muscle fiber type from anaerobic to aerobic myofibers *via* stimulation of the FGF21-sirtuin type 1 (SIRT1)-AMPK-PPAR g coactivator 1a (PGC1α) axis *in vitro* on C2C12 cells and *in vivo* on skeletal muscle-specific FGF21 transgenic mice (Liu et al., 2017). It is therefore of interest to know if muscle-derived FGF21 plays a role in muscle fiber type switching under pathological conditions that are accompanied by elevated FGF21 expression from skeletal muscle, and whether the resulting muscle fiber type is an adaptive or causative response to the primary pathology.

Mitochondria are critical for cellular energy generation and biosynthetic pathways and regulation of their function is paramount for muscle physiology and metabolism (Hood et al., 2019). Multiple studies have demonstrated enhanced mitochondrial biogenesis and oxidative functions in both liver and adipose tissue under the treatment of FGF21. Specifically, FGF21 regulates mitochondrial oxidative function in adipocytes *via* the AMPK-SIRT1-PGC1α pathway (Chau et al., 2010). In liver, pathways involved in activation of PGC1α were also observed in FGF21 transgenic mice and WT mice treated with FGF21 (Potthoff et al., 2009; Fisher et al., 2011). However, if a similar signaling pathway is also present in skeletal muscle remains unknown. Since FGF21 is induced in and secreted from skeletal muscle in mitochondrial myopathies and various mitochondrial stressors, it might also act as an adaptive mediator of the muscle mitochondrial stress *via* activation of pathways that control mitochondrial function (Oost et al., 2019, 2020; Klaus and Ost, 2020). It would be of interest to determine whether FGF21 also regulates mitochondrial biogenesis and function in muscle *via* activation of AMPK, SIRT1, and PGC1α.

## The Effects of FGF21 on Bone

Whether FGF21 has a positive or detrimental effect on bone in mice and humans remains unclear. Studies have shown that FGF21-Tg mice and mice administrated recombinant FGF21 have reduced bone mass (Wei et al., 2012; Zhang et al., 2012). Increased serum FGF21 has also been reported to negatively affect bone mineral density (BMD) during lactation in C57BL/6 mice (Bornstein et al., 2014). Moreover, in a mouse model of DMD, which shows elevated circulating skeletal muscle-derived FGF21 (Zhou et al., 2018), blockage of FGF21’s action using a neutralization antibody, resulted in significantly increased bone mass and improved quality of bone tissues (Li et al., 2020). However, conflicting data exists as others have reported no bone loss observed in FGF21 KO mice nor in recombinant FGF21 treated mice (Li et al., 2017). Additionally, no significant bone loss was detected in AAV8-hAAT-FGF-21 genetically engineered high fat diet-induced obese mice (Jimenez et al., 2018).

Inconsistencies are also found in human studies. Plasma FGF21 concentrations have been shown to negatively correlate with femoral neck BMD in a Han Chinese adult population (Hao et al., 2018) and in healthy aged adults (Lee et al., 2020). Serum FGF21 levels were found to be associated with worsened radial trabecular bone microarchitecture and decreased radial bone strength in women with anorexia nervosa (Fazeli et al., 2015a). Elevated FGF21 levels are also associated with poor bone health in HIV-1 infected patients (Gallego-Escuredo et al., 2017). Obese humans and non-human primates administered a long-acting FGF21 analog demonstrated increased plasma biomarkers of bone resorption and decreased bone formation, which indicates bone loss (Talukdar et al., 2016; Kim et al., 2017). On the other hand, others have reported no clear correlation of serum FGF21 with BMD and fragility fractures (Choi et al., 2018; Hu et al., 2018). Furthermore, an independent positive association between plasma FGF21 levels and BMD in 40 healthy young women has also been reported (Lee et al., 2013). The inconsistency may be due to the heterogeneous populations and disease states; the effects of FGF21 on bone health are likely duration and context-dependent. Future studies should consider the influence of underlying diseases.

Although the exact mechanism of how FGF21 regulates bone homeostasis is still not clear, several direct and indirect mechanisms have been proposed (Figure 2). The expression of FGFRs/β-klotho in bone tissue has not been fully established, however, a recent study on dystrophic mice demonstrated that β-klotho and FGFR expression is significantly induced in mature osteoclasts (Li et al., 2020). These data indicate that bone is a direct target of FGF21 and FGF21 may affect osteoclastogenesis in DMD. In addition to the direct effects, accumulating evidence also suggests indirect effects of FGF21 on bone. Studies have shown direct stimulation of adipogenesis in bone marrow-derived mesenchymal stem cells by FGF21 (Wei et al., 2012; Li et al., 2020). FGF21 has close connections with the somatotropic axis, which plays an important role in protein synthesis and bone homeostasis (Milman et al., 2016). Transgenic mice overexpressing FGF21 showed evidence of growth hormone (GH) resistance in the liver, which significantly reduced the level of serum insulin-like growth factor-1 (IGF-1; Inagaki et al., 2008; Kubicky et al., 2012). FGF21 blunts hepatic GH signaling *via* inhibition of STAT5 signaling, increased expression of IGF-1 binding protein 1 (IGFBP-1), and increased expression of suppressor of cytokine signaling 2 (SOCS2; Inagaki et al., 2008). In addition, FGF21 inhibits GH’s action on proliferation and differentiation of chondrocytes directly at the growth plate (Wu et al., 2012, 2013). Moreover, Wei et al. (2012) reported that FGF21 promotes IGFBP-1 release from liver, which consequently enhanced osteoclastogenesis and provoked bone resorption. Interestingly, elevated IGFBP-1 was not observed in DMD animal models (Li et al., 2020) and in aged populations (Lee et al., 2020), both of which demonstrate elevated serum FGF21 and pathologic bone changes.

Although the clinical data are still controversial and further studies are needed to address the molecular mechanisms of FGF-21’s action on bone, the current literature suggests adverse effects of FGF21 on bones, which needs to be carefully addressed in future human studies.

## Discrepancies Between Mice and Humans

An abundance of data has been generated in mice and revealed several mechanistic findings with regards to FGF21 expression, regulation, and function. However, as literature regarding FGF21 analysis in humans has expanded, differences between mice and humans have been identified and recently reviewed (Staiger et al., 2017; Keuper et al., 2020). The discrepancies include circulating FGF21 levels, tissue-specific expression and regulation, and its role in glucose and lipid metabolism in addition to metabolic diseases.

Serum levels of FGF21 not only vary significantly between mice and humans but within the species themselves. In mice, serum FGF21 concentrations in chow-fed mice range from 0.1 to 3,000ng/ml (Badman et al., 2007; Fisher et al., 2010; Dutchak et al., 2012; Murata et al., 2013; Tezze et al., 2017; Jimenez et al., 2018). Further complicating data interpretation, variations in mouse serum FGF21 are dependent on the strain tested, animal age, phase of the circadian cycle, and the assay used to quantify serum levels (Tezze et al., 2019). Wide variability also exists in healthy humans with published serum concentrations of FGF21 ranging from 21 to 7,100pg/ml (Galman et al., 2008; Zhang et al., 2008; Li et al., 2009; Dushay et al., 2010; Kralisch et al., 2013; Fazeli et al., 2015b).

While FGF21 gene expression in murine models occurs in the liver, pancreas, adipose tissue, skeletal muscle, and other tissues in the basal state (Nishimura et al., 2000; Itoh, 2014; Petryszak et al., 2016), our current understanding is that the liver expresses and releases into the circulation the majority of FGF21 in healthy humans (Dushay et al., 2010; Petryszak et al., 2016; Keuper et al., 2020). However, it should be noted that most *in vivo* human data are correlational in nature, lacking mechanistic insights.

With regards to the regulation of FGF21 in mice and humans, protein restriction, fructose ingestion, exercise, and circadian clock machinery all induce hepatic expression and elevated circulating levels of FGF21 in both species (Staiger et al., 2017). Similarities also exist with the stimulation of FGF21 from skeletal muscle during conditions of muscle-specific mitochondrial disease, certain types of exercise, and hyperinsulinemia in mice and humans (Hojman et al., 2009; Crooks et al., 2014; Emanuelli et al., 2014; Tanimura et al., 2016; Staiger et al., 2017). As previously discussed, inter-species discrepancies exist with regards to the impact of nutritional stressors on circulating and muscle-derived FGF21. Nutrient deprivation/fasting and consumption of ketogenic diets result in a rapid rise of FGF21 serum levels in mice (Badman et al., 2007), however, elevations of FGF21 are only seen in humans after prolonged fasting period of at least 7days (Galman et al., 2008; Fazeli et al., 2015b). Moreover, differences also exist between species in response to hormone inducers. In mouse models, studies have shown an increase in hepatic-derived serum FGF21 levels in response to GH administration (Chen et al., 2011) and thyroid hormone (Adams et al., 2010; Domouzoglou et al., 2014), however, no acute effect on serum FGF21 was seen in humans in response to GH or thyroid hormone administration (Lundberg et al., 2013; Bonde et al., 2014).

Although caution should be taken when translating murine study results to humans, mouse models of FGF21 expression serve an invaluable role in elucidating the complex interplay of this hormone in human physiology and disease.

## Conclusion and Future Perspectives

Our understanding of the role of FGF21 in biological systems has advanced enormously over the last two decades. Although the main function of FGF21 as a starvation-induced hormone secreted from the liver has been known and broadly understood for some time, the ectopic expression and secretion of FGF21 from skeletal muscle and its potential role in a range of mitochondrial and muscular disorders is increasingly recognized. There is compelling evidence to indicate novel effects of FGF21 on skeletal muscle and bone, however, many outstanding questions remain. (1) Although many studies have observed and reported the phenotypic changes in skeletal muscle and bone in the presence of elevated FGF21, compared to our understanding of the function of FGF21 on liver and adipose tissue, the mechanism(s) of its action on skeletal muscle and bone is still largely unknown. More work is required to elucidate the cellular and molecular mechanisms of muscle-derived FGF21 and to understand its regulation and function at the whole-body level. (2) Clinical validation of FGF21 as a biomarker of disease and potential therapeutic target in mitochondrial myopathies and DMD is still needed. (3) With the unraveling of the cross-interactions with other signaling pathways, such as AMPK-SIRT1 (Chau et al., 2010), GH (Inagaki et al., 2008; Wu et al., 2012), and glucocorticoids (Patel et al., 2015), the effects of muscle-derived FGF21 might be pathological context-dependent. Our understanding of the pathologic role and even the pharmacologic effects of FGF21 need to be more disease/condition specific. (4) Additionally, although some mechanisms have been proposed, the exact cellular and molecular mechanisms of FGF21 secretion from skeletal muscle under different pathological conditions and the target cells within the musculoskeletal system, specifically skeletal muscle and bone, are still largely unknown. A promising approach towards understanding the mechanism of FGF21 includes continued development of tissue-specific FGF21 and/or β-klotho receptor/KO animal models. Furthermore, tissue-specific receptor and/or KO animal models based on specific disease conditions will also be needed to understand the role of FGF21 under a specific pathological context.

## Author Contributions

HS and MS performed reference analysis and manuscript preparation. HL designed research (project conception, development of overall research plan, and oversight) and had primary responsibility for final content, reference analysis, and manuscript preparation. All authors contributed to the article and approved the submitted version.

### Conflict of Interest

The authors declare that the research was conducted in the absence of any commercial or financial relationships that could be construed as a potential conflict of interest.
